# LM02 trial Perioperative treatment with panitumumab and FOLFIRI in patients with wild-type RAS, potentially resectable colorectal cancer liver metastases—a phase II study

**DOI:** 10.3389/fonc.2023.1231600

**Published:** 2023-08-09

**Authors:** Gudrun Piringer, Thomas Gruenberger, Josef Thaler, Irene Kührer, Klaus Kaczirek, Friedrich Längle, Istvan Viragos-Toth, Arno Amann, Wolfgang Eisterer, Reinhold Függer, Johannes Andel, Angelika Pichler, Judith Stift, Lidija Sölkner, Michael Gnant, Dietmar Öfner

**Affiliations:** ^1^ Department of Internal Medicine IV, Klinikum Wels-Grieskirchen, Wels, Austria; ^2^ Medical Faculty, Johannes Kepler University Linz, Linz, Austria; ^3^ Department of Surgery, Clinic Favoriten, Hepato-Pancreato-Biliary Center, Health Network Vienna and Sigmund Freud University, Vienna, Austria; ^4^ Division of General Surgery, Department of Surgery, Medical University of Vienna, Vienna, Austria; ^5^ Department of Surgery, Landesklinikum Wiener Neustadt, Wiener Neustadt, Austria; ^6^ Department of Haematology and Oncology, Medical University of Innsbruck, Innsbruck, Austria; ^7^ Department of Internal Medicine and Oncology, Klinikum Klagenfurt, Klagenfurt, Austria; ^8^ Department of General and Visceral Surgery, Congregation Hospital, Linz, Austria; ^9^ Department of Internal Medicine II, Landeskrankenhaus Steyr, Steyr, Austria; ^10^ Department of Hematology and Oncology, Landeskrankenhaus Hochsteiermark, Leoben, Austria; ^11^ Department of Pathology, Medical University of Vienna, Vienna, Austria; ^12^ Department of Statistics, Austrian Breast and Colorectal Cancer Study Group, Vienna, Austria; ^13^ Comprehensive Cancer Center, Medical University of Vienna, Vienna, Austria; ^14^ Department of Visceral-, Transplant- and Thoracic Surgery, Medical University of Innsbruck, Innsbruck, Austria

**Keywords:** LM02-trial, perioperative therapy, CRLM, panitumumab-FOLFIRI, anti-EGFR-therapy

## Abstract

**Background:**

Twenty percent of colorectal cancer liver metastases (CLMs) are initially resectable with a 5-year survival rate of 25%–40%. Perioperative folinic acid, 5-fluorouracil, oxaliplatin (FOLFOX) increases progression-free survival (PFS). In advanced disease, the addition of targeting therapies results in an overall survival (OS) advantage. The aim of this study was to evaluate panitumumab and FOLFIRI as perioperative therapy in resectable CLM.

**Methods:**

Patients with previously untreated, wild-type Rat sarcoma virus (RAS), and resectable CLM were included. Preoperative four and postoperative eight cycles of panitumumab and folinic acid, 5-fluorouracil, irinotecan (FOLFIRI) were administered. Primary objectives were efficacy and safety. Secondary endpoints included PFS and OS.

**Results:**

We enrolled 36 patients in seven centers in Austria (intention-to-treat analyses, 35 patients). There were 28 men and seven women, and the median age was 66 years. About 91.4% completed preoperative therapy and 82.9% underwent liver resection. The R0 resection rate was 82.7%. Twenty patients started and 12 patients completed postoperative chemotherapy. The objective radiological response rate after preoperative therapy was 65.7%. About 20% and 5.7% of patients had stable disease and progressive disease, respectively. The most common grade 3 adverse events were diarrhea, rash, and leukopenia during preoperative therapy. One patient died because of sepsis, and one had a pulmonary embolism grade 4. After surgery, two patients died because of hepatic failure. Most common grade 3 adverse events during postoperative therapy were skin toxicities/rash and leukopenia/neutropenia, and the two grade 4 adverse events were stroke and intestinal obstruction. Median PFS was 13.2 months. The OS rate at 12 and 24 months were 85.6% and 73.3%, respectively.

**Conclusions:**

Panitumumab and FOLFIRI as perioperative therapy for resectable CLM result in a radiological objective response rate in 65.7% of patients with a manageable grade 3 diarrhea rate of 14.3%. Median PFS was 13.2 months, and the 24-month OS rate was 73.3%. These data are insufficient to widen the indication of panitumumab from the unresectable setting to the setting of resectable CLM.

## Introduction

Colorectal cancer is the second leading cause of mortality in Western countries (1, 2). Nearly half of patients will develop colorectal cancer liver metastases (CLMs) during the course of their disease, with 15%–25% of patients having CLM at the primary diagnosis and another 20% of patients will develop CLM during the first 3 years after the primary diagnosis (3, 4). About one-fifth of patients with CLM have no other sites of metastasis. Despite advances in survival with chemotherapy, surgical resection of CLM is still considered the only curative treatment option. About 20% of patients with CLM are candidates for primary resection (5) and result in a 25%–40% 5-year survival (6–9). Unfortunately, 70% of patients will develop recurrent disease after liver resection (10).

The advantages of postoperative chemotherapy after curative resection of CLM resection are uncertain. In the European Organisation for Research and Treatment of Cancer (EORTC) 40983 study, perioperative chemotherapy with FOLFOX4 and surgery were compared with surgery alone in patients with potentially resectable CLM (11). Progression-free survival (PFS) was significantly improved by 9.2% at 3 years for those who received perioperative chemotherapy. However, the trial did not demonstrate any significant benefit in overall survival (OS) (12). Similar results were shown in a meta-analysis evaluating perioperative chemotherapy for patients with resectable CLM (13). The observed benefit in PFS with perioperative FOLFOX remains one of the standard treatments for resectable CLM in many centers worldwide.

The addition of targeting therapies to chemotherapy has markedly improved outcome in metastatic colorectal cancer (mCRC) and significantly improves the objective response rate (ORR), PFS, and OS (14–20). Furthermore, combination therapies may convert unresectable to resectable liver metastases, allowing potentially curative resection.

The optimal combination of systemic drugs in the neoadjuvant setting of patients with potentially resectable CLM has not been established. Unanswered questions are the best chemotherapy combinations with or without targeted agents to induce maximum response, the length of initial treatment to verify response without liver tissue damage, and the correlation of response with potential biomarkers.

The present LM02 trial from the Austrian Breast and Colorectal Cancer Study Group (ABCSG) investigated the use of perioperative systemic therapy with panitumumab and FOLFIRI in patients with primary resectable CLM.

## Methods

### Patient population

Patients with wild-type RAS mCRC with potentially histologically confirmed resectable liver metastases, at least one measurable metastatic lesion in the liver as per the Response Evaluation Criteria in Solid Tumor (RECIST) 1.1 guidelines, and without prior therapy for mCRC were eligible. CLMs were defined as resectable when it was anticipated that the disease can be completely resected, two adjacent liver segments could be spared adequate vascular inflow and outflow and biliary drainage could be preserved, and the volume of the liver remaining after resection would be adequate (at least 20% of the total estimated liver volume). Other key eligibility criteria included: patients ≥ 18 years with Eastern Cooperative Oncology Group (ECOG) performance status 0 and 1, and adequate metabolic, hematological, renal, and hepatic functions. We excluded patients with (a) prior chemotherapy for the treatment of current mCRC including biologics; (b) extrahepatic metastatic disease; (c) prior adjuvant or neoadjuvant (chemo)therapy for the treatment of CRC ≤ 26 weeks prior to registration; (d) radiotherapy ≤ 14 days prior to registration; (e) previous malignancy other than CRC in the last 5 years except basal cell carcinoma of the skin and/or *in situ* carcinoma of the cervix; (f) active infection requiring systemic treatment; (g) any investigational agent or therapy ≤ 28 days before registration; (h) clinically significant cardiovascular disease ≤ 1 year before registration; (i) known allergy or hypersensitivity to irinotecan; 5-fluorouracil (5-FU), leucovorin, or panitumumab; (j) history of severe adverse events (AEs) to iodinated contrast agents; (k) history of interstitial pneumonitis or pulmonary fibrosis or evidence of interstitial lung disease on baseline chest computer tomography (CT) scan; (l) known positive test(s) for human immunodeficiency virus infection, hepatitis C virus, and acute or chronic active hepatitis B infection; (m) any co-morbid disease or condition that could increase the risk of toxicity; (n) any uncontrolled concurrent illness or history of any medical condition that may interfere with the interpretation of the study results; (o) major surgical procedure (requiring general anesthesia) ≤ 28 days prior registration and (p) pregnant or breastfeeding women.

Exploratory biomarker studies suggested that other activating RAS mutations also were a negative predictive biomarker for anti-epithelial growth factor receptor (EGFR) therapy. Patients with mutations beyond the known K-Rat sarcoma virus (KRAS) exon 2 mutations, in KRAS exon 3 (codons 59 and 61), exon 4 (codons 117 and 146) or NRAS exon 2 (codons 12 and 13), exon 3 (codons 59 and 61), and exon 4 (codons 117 and 146), did not respond to anti-EGFR therapy. These data were published during the running study (15, 21, 22). Therefore, the study was stopped in 2013 temporarily for an amendment. In the first phase, patients with wild-type KRAS were included, and, after the amendment, only patients with wild-type RAS mCRC were included. Microsatellite status was not evaluated because the importance of Microsatellite instability status (MSI) was not known in the recruitment period.

Medical ethics committees of all participating centers approved the trial, and all patients provided a written informed consent (EudraCT-No: 2012_000265-20). This study was sponsored by the ABCSG.

### Study design

This was an open-label phase II multicenter trial to evaluate the efficacy and safety of perioperative panitumumab in combination with FOLFIRI and liver resection in patients with previously untreated, wild-type RAS, and potentially resectable CLM. The preoperative therapy consisted of four cycles panitumumab and FOLFIRI every 14 days. Surgery was performed 4–8 weeks after the last administration of the study medication. Postoperative eight cycles of panitumumab and FOLFIRI were planned 4–6 weeks after surgery. Follow-up was done for up to 2 years after the end of postoperative chemotherapy. A uniform CT with liver protocol was done in all seven sites. PET-CT was not standard imaging. Primary endpoints were ORR and the rate of grade 3–4 diarrhea during preoperative therapy. Secondary endpoints included evaluation of resection rate, perioperative morbidity and mortality as measured by the Dindo classification (23), proportion of subjects with complete pathological response as measured by Rubbia–Brandt tumor regression grade (24), PFS, and OS. The study was a two-step design. After application of preoperative therapy to 15 patients, safety and efficacy were evaluated. If at least five patients exhibited an objective response according to RECIST 1.1 and if there were not more than three patients with grade 3–4 diarrhea during the four cycles of therapy, then additional 21 patients were included.

### Treatment

#### Preoperative treatment

Patients were treated with four cycles panitumumab and FOLFIRI before surgery of the liver metastases. Panitumumab was administered at a dose of 6 mg/kg on day 1 of each cycle. Irinotecan of 180 mg/m^2^ was administered followed by leucovorin 400 mg/m^2^. Thereafter, 5-FU of 400mg/m^2^ was given as an intravenous bolus followed by a 5-FU continuous intravenous infusion of 2,400 mg/m^2^ over 46 h. A cycle of panitumumab and FOLFIRI was defined as 14 days. Toxicities were assessed and recorded at every visit and graded according to the common terminology criteria for AEs (National Cancer Institute common Terminology Criteria for Adverse Events (NCI CTCAE) version 4.0) (25). Panitumumab and FOLFIRI dose modification schemes were applied if patients experienced grade 2–4 toxicities. Dose modification was not required for toxicities that were considered unlikely to become serious or life-threatening (e.g., alopecia).

#### Surgery

After end of preoperative therapy, chest–abdomen–pelvis CT and tumor marker to access response to preoperative therapy was carried out 2–3 weeks after last administration of the study medications. Surgery was planned 4–8 weeks after end of therapy and was performed by experienced liver surgeons. Synchronous resection of liver metastases and primary tumor was allowed if liver resection was limited to <2 liver segments. Otherwise, primary tumor was resected 4–6 weeks after liver resection. In case the tumor deemed non-resectable, following therapy according to the institutional standard was recommended.

#### Postoperative treatment

Postoperative eight cycles of panitumumab and FOLFIRI were planned every 14 days. Therapy was started 4–6 weeks after last surgery following CT assessment and complete wound healing.

### Statistical analysis

The primary endpoints ORR and safety were evaluated descriptively. ORR after four cycles of preoperative therapy was evaluated using RECIST 1.1 measured by multislice three-phase CT. To assess safety the rate of patients with grade 3–4 diarrhea during four cycles of preoperative therapy was documented. The sample size for the primary endpoints was estimated using the Bryant & Day Phase II clinical trial design. Because of the two-step design, the safety and efficacy after application of preoperative therapy to 15 patients were evaluated. If at least five patients exhibited an objective response according and if there were not more than three patients with grade 3–4 diarrhea during these four cycles, then additional 21 patients would be included. The statistical analysis was based on the “intention-to-treat” (ITT) principle, all patients enrolled were included. Patients who withdraw informed consent were considered as non-successes (without ORR and with grade 3–4 diarrhea). Patients who would not undergo surgical resection were included in the final analyses. Evaluation of secondary endpoints included resection rate, perioperative morbidity and mortality, rate of complete pathological remission, PFS, and OS.

## Results

**Figure 1:**
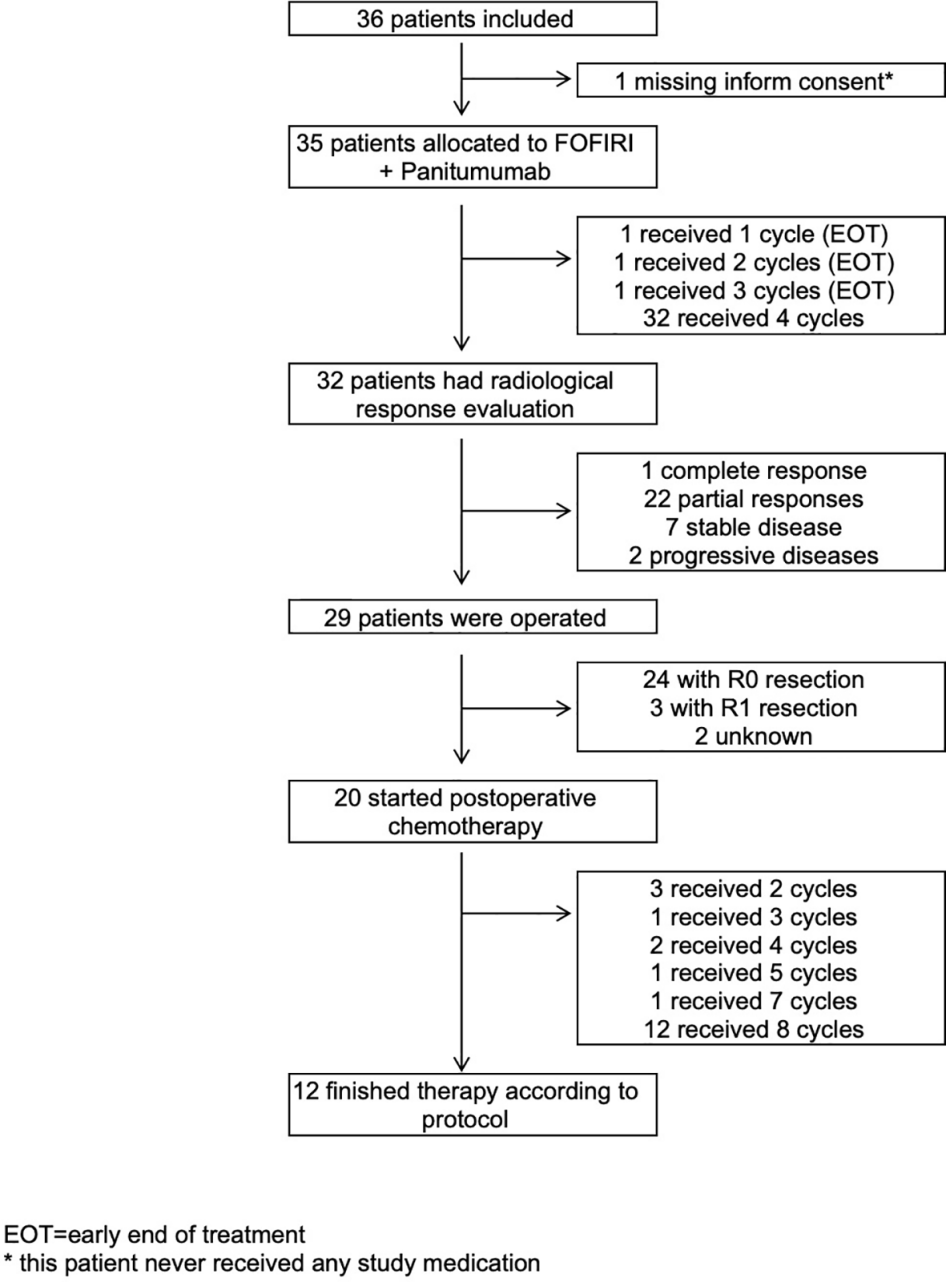
Trial profile. EOT, early end of treatment. * This patient never received any study medication.

A total of 36 patients were enrolled into the study in seven Austrian institutions from October 2012 through June 2017. ITT analyses included 35 patients (**Figure 1**). One patient was excluded because the informed consent was missing. Nevertheless, this patient never received any study medication due to an exclusion criterion (hepatitis C virus). Of the patients, 28 (80%) were men and seven (20%) were women. The median age was 66 years (range, 32–81). A total of 19, 6, and 10 patients had colon cancer, cancer in the rectosigmoid, and rectal cancer, respectively. Of the 35 patients, 20 (57.1%) and 15 (42.9%) developed liver metastases metachronous and synchronous at primary tumor diagnosis, respectively. Liver metastases occurred in 23 patients (65.7%) within 2 years of primary cancer diagnoses and in nine patients (25.7%) after 2 years. Most of patients had one liver metastases (n = 13, 37.1%), followed by two lesions in 10 patients (28.6%); three lesions in six patients (17.1%); and four, five, and six lesions each in two patients (5.7%). ECOG performance status 0 and 1 was in 29 (82.9%) and six (17.1%) patients. Fifteen (42.9%) patients had wild-type KRAS tumor, and 20 (57.1%) patients had wild-type RAS tumor. In seven (20%) patients, radiotherapy of the primary rectal tumor was done. In 25 (71.4%) and eight (22.9%) patients, primary tumors were resected before registration for the study and during study, and two (5.7%) patients had no surgery of the primary tumor. Adjuvant chemotherapy for the primary cancer was administered to seven patients (20%). **Table 1** shows the baseline characteristics of the patients.

**Table 1:** Characteristics of patients at baseline.

Age (years)	
Median (range)	66 (32–81)
Sex	
Men Women	28 (80%) 7 (20%)
WHO performance status	
0 1	29 (82.9%) 6 (17.1%)
Synchronicity of liver metastases	
Synchronous metastases Metachronous metastases	15 (42.9%) 20 (57.1%)
Time from diagnosis of primary to diagnosis of liver metastases	
< 2 years > 2 years	23 (65.7%) 9 (25.7%)
Number of liver metastases	
1 2 3 4 5 6	13 (37.1%) 10 (28.6%) 6 (17.1%) 2 (5.7%) 2 (5.7%) 2 (5.7%)
T category of the primary cancer	
T1 T2 T3 Tx	1 (2.9%) 5 (14.3%) 21 (60.0%) 8 (22.9%)
Lymphatic spread of the primary cancer	
N0 N1 N2 Nx	11 (31.4%) 8 (22.9%) 5 (14.3%) 11 (31.4%)
Location of primary cancer	
Colon Rectosigmoid Rectum	19 (54.3%) 6 (17.1%) 10 (28.6%)
Previous adjuvant chemotherapy of primary cancer	
No Yes	28 (80%) 7 (20%)

Efficacy and safety were evaluated after 15 patients according to the two-step design of the trial. Objective response was found in 13 (86.7%) of the 15 patients with 12 partial remissions (PRs) and one complete response (CR). One patient had a stable disease (SD), and one patient had missing response evaluation. Grade 3 diarrhea occurred in two (13.3%) patients, and no grade 4 diarrhea was observed during the preoperative therapy. On the basis of these results, the criteria for continuing the study were met.

In the final analysis, 32 (91.4%) of the 35 patients completed the planned four cycles of preoperative therapy. One patient each had stopped preoperative therapy with panitumumab and FOLFIRI after the first, second, and third cycle. In the preoperative treatment phase, 34 (97.1%) patients suffered at least one grade 1–5 AE. Thirteen (37.1%) patients had at least one grade 3 AE, one patient (2.9%) had grade 4 AE (pulmonary embolism), and one patient (2.9%) had grade 5 AE (sepsis). Most grade 3 AEs were diarrhea in four patients (11.4%), leukopenia in three patients (8.6%), rash in three patients (8.6%), acne in two patients (5.7%), and one patient each (2.9%) had cardiac failure, pyrexia, urinary tract infection, uncontrolled hypertension, dehydration, stroke, dyspnea, and maculo-papular skin toxicity as grade 3 AEs (**Table 2**). Grade 3 diarrhea during preoperative therapy occurred in four patients. In one patient who discontinued treatment early due to AEs, the documentation whether he had a grade 3–4 diarrhea was missing. Therefore, the occurrence of a diarrhea grade 3–4 AE was assumed in accordance with a worst-case scenario for the main ITT analysis. Hence, diarrhea grade 3 occurred in 14.3% of patients.

**Table 2:** Adverse events during preoperative and postoperative therapy.

Adverse Event	Grade 3	Grade 4	Grade 5
During preoperative chemotherapy period			
Leukopenia Cardiac failure Diarrhoe Pyrexia Urinary tract infection Blood pressure increased Dehydration Cerebrovascular stroke Dyspnoe Pulmonary embolism Rash/Acne/Dermatitis Sepsis	3 (8.6%) 1 (2.9%) 4 (11.4%) 1 (2.9%) 1 (2.9%) 1 (2.9%) 1 (2.9%) 1 (2.9%) 1 (2.9%) 6 (17.1%)	1 (2.9%)	1 (2.9%)
During postoperative chemotherapy period			
Anaemia Leukopenia/Neutropenia Diarrhoea Ileus Anal Abscess fungal infection cerebrovascular stroke Pulmonary embolism rash/skin toxicity deep vein thrombosis	1 (5%) 2 (10%) 1 (5%) 1 (5%) 1 (5%) 1 (5%) 4 (20%) 1 (5%)	1 (5%) 1 (5%)	

Data in number (%) unless otherwise stated. Patients may have several complications; therefore, the number of complications does not add up to total number of patients. Common toxicity criteria (25) version 4.0 was used.

Among 35 patients in the ITT population, 32 (91.4%) had a documented radiological response evaluation after preoperative therapy (**Table 3**). Those three patients without a documented response evaluation discontinued treatment early due to a serious AE or investigators decision and died shortly after treatment discontinuation. According to ITT rules (worst case), their objective response was evaluated as “no objective response.” Objective radiological response rate after preoperative therapy was 65.7% (n = 23) with one radiological complete remission (CR) (2.7%) and 22 (62.7%) PR. In 20% (n = 7) and 5.7% (n = 2) of patients, SD and progressive disease (PD) were documented, respectively. A sensitivity analysis, only in patients who finished all four cycles of preoperative therapy, resulted in an ORR of 71.9%.

**Table 3:** Response to preoperative therapy according to RECIST.

Excluded from response analysis	3 (8.6%)
EOT after one cycle EOT after two cycles EOT after three cycles	1 (2.9%) 1 (2.9%) 1 (2.9%)
Response evaluation	32 (91.4%)
Complete response Partial response Stable disease Progressive disease	1 (2.9%) 22 (62.9%) 7 (20.0%) 2 (5.7%)
Sums of largest diameters of target lesions on imaging *	
At baseline (mm) After preoperative therapy (mm) Relative reduction (%)	1,770.5 mm 1,019.5 mm 42.4%

*Measured in all 32 patients with imaging at baseline and with response evaluation.

EOT, early end of treatment.

Surgery of the liver metastases was done in 29 (82.9%) patients (**Table 4**). Surgery was done after a mean of 6.62 weeks (median, 6.14 weeks; range, 3–11.9 weeks) after last administration of preoperative therapy. Reasons for non-resection were early end of treatment (EOT) during preoperative phase in three patients, documented progression disease with new liver lesions in one patient, inadequate future liver remaining in one patient (despite a PR after preoperative therapy), and inoperable retrospective at baseline in one patient. R0 and R1 resection rate was 82.8% (n = 24) and 10.3% (n = 3). In one patient, the resection rate was not measurable, and, in one patient, the documentation of resection rate was missing. Types of liver resection are shown in **Table 4**. Histological tumor response to preoperative therapy was centrally evaluated using the Rubbia–Brandt classification (**Table 5**) (24). From 29 patients with surgery, in four patients evaluation was not possible. There were two patients with Rubbia–Brandt tumor regression grade 1 (complete pathological response). Two patients died after surgery because of hepatic failure: one patient within 30 days after surgery and the other one 120 days after surgery (**Table 4**). Both patients had a hemihepatectomy right. One patient had SD, and one patient PR after preoperative therapy. The sum size of metastases was 112 and 75 mm. Surgical complications were measured by the Clavien–Dindo classification (**Table 6**).

**Table 4:** Surgical information and postoperative complications.

Operated (number of resected liver metastases)	29 (82.9%)
1 2 3 4 5 7 8 9	12 (41.4%) 7 (24.1%) 1 (3.5%) 3 (10.3%) 2 (6.9%) 2 (6.9%) 1 (3.5%) 1 (3.5%)
Overview of type of liver resection	
Atypical resection (Bi)segmentomy Left hemihepatectomy Right hemihepatectomy Radiofrequency ablation	29 (51%) 9 (16%) 3 (5%) 12 (21%) 4 (7%)
Not operated	6 (17.1%)
EOT during preoperative phase Progressive disease Inadequate future liver remnant Inoperable at baseline in retrospect	3 (8.6%) 1 (2.9%) 1 (2.9%) 1(2.9%)
Tumor on specimen from resection	
Macroscopic Only microscopic residual No residual tumor Missing	0 3 (10.3%) 24 (82.8%) 2 (6.9%)
At least one major postoperative complication	
Death during surgery Postoperative death < 30 days after liver resection > 90 days after liver resection Suture related complication grade 3	0 2 (6.9%) 1 (3.4%) 1 (3.4%) 1 (3.4%)

EOT, early end of treatment. Data in number (%) unless otherwise stated.

**Table 5:** Rubbia–Brandt tumor regression grade.

Rubbia–Brandt tumor regression grade	Frequency	Percent
Missing (no surgery)	6	17.14
Grade 1	2	5.71
Grade 2	5	14.29
Grade 3	4	11.43
Grade 4	11	31.43
Grade 5	3	8.57
n.b. (not evaluable)	4	11.43

**Table 6:** Clavien–Dindo classification.

Clavien–Dindo classification grade	Frequency	Percent
Grade I	22	78.57
Grade II	3	10.71
Grade IIIB	1	3.57
Grade IVa	1	3.57
Grade V	1	3.57

Twenty (57.1%) patients started postoperative chemotherapy, of whom 12 (60%) received eight cycles. In nine patients, postoperative therapy was not started because of AEs in the preoperative/postoperative phase in four patients (severe acneiforme dermatitis in two patients and postoperative death in two patients), no response or progression in the preoperative phase in three patients, and investigators decision in two patients (one patient was non-compliant and one due to secondary carcinoma). **Table 2** shows the tolerance to postoperative therapy.

**Figure 2:**
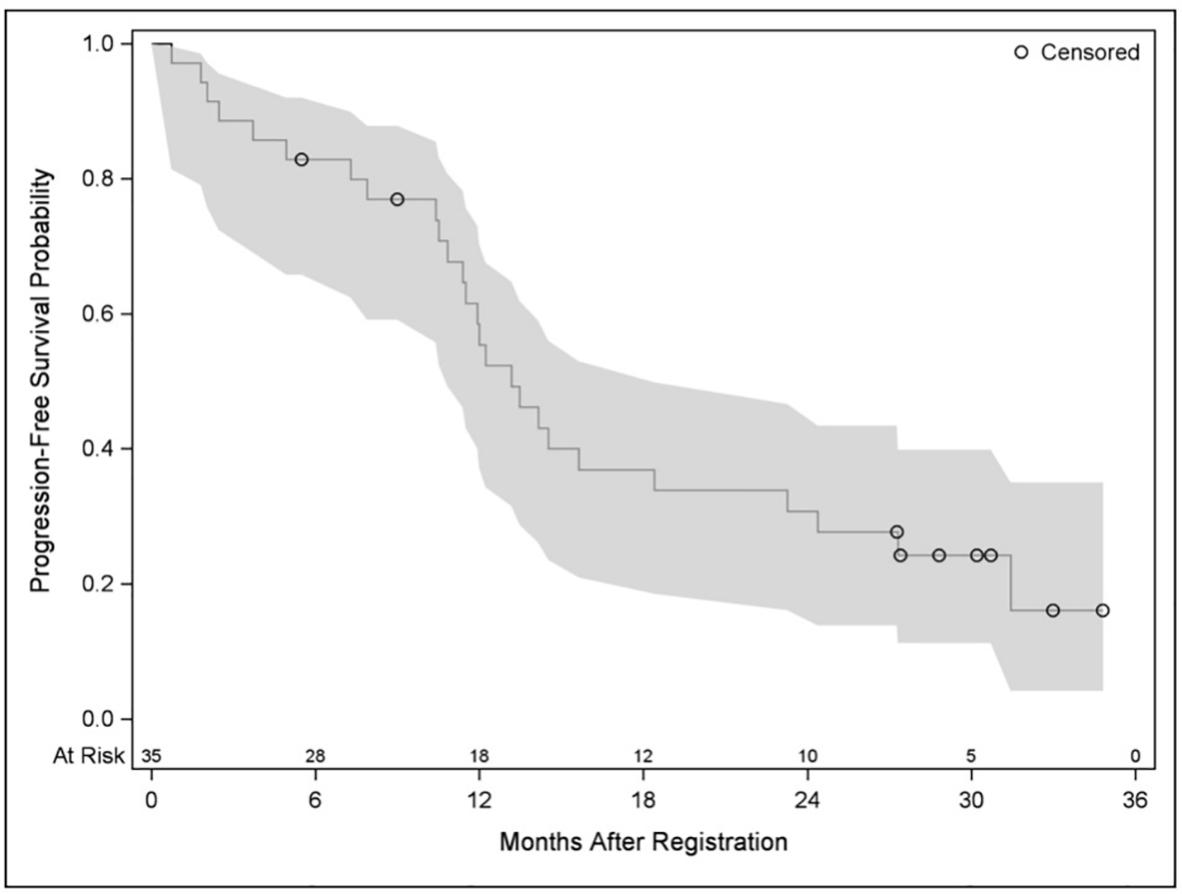
Kaplan–Meier plot for progression-free survival.

**Figure 3:**
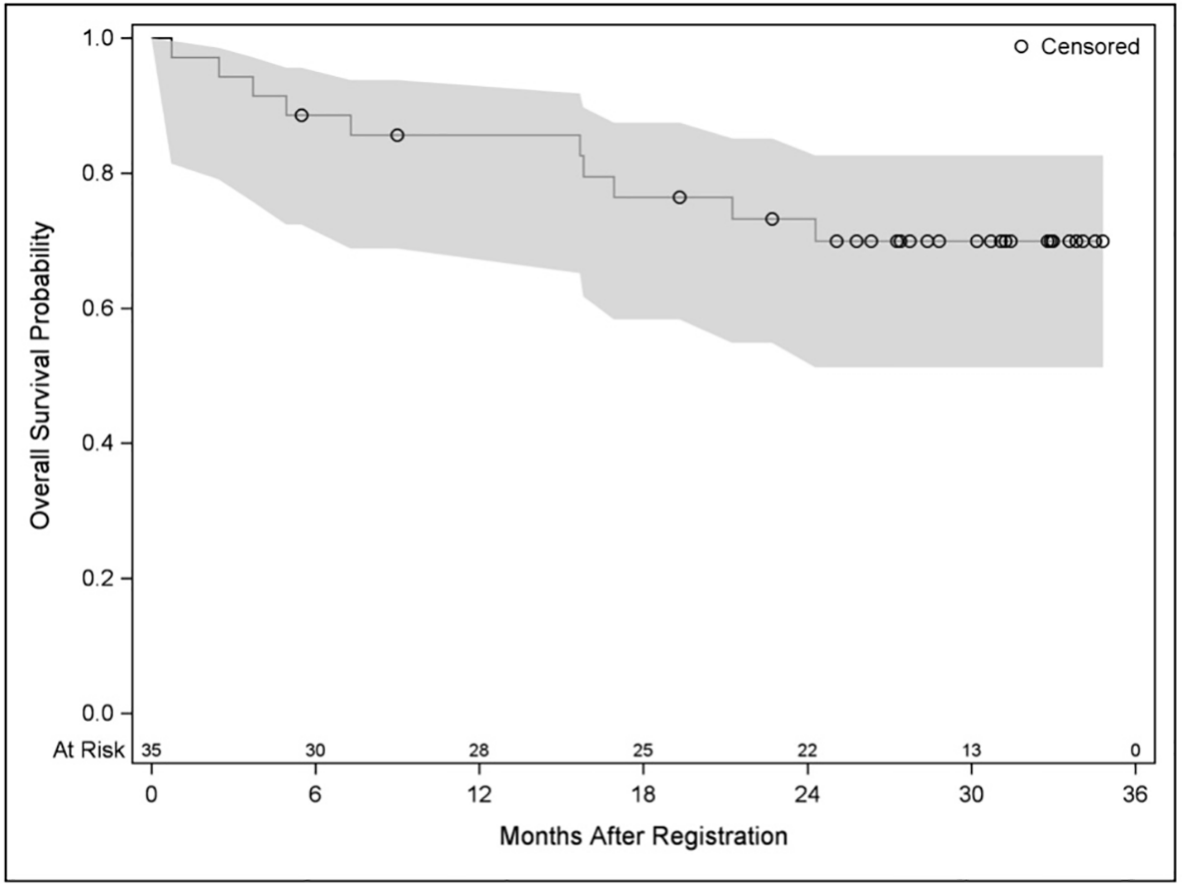
Kaplan–Meier plot for OS.

**Figure 4:**
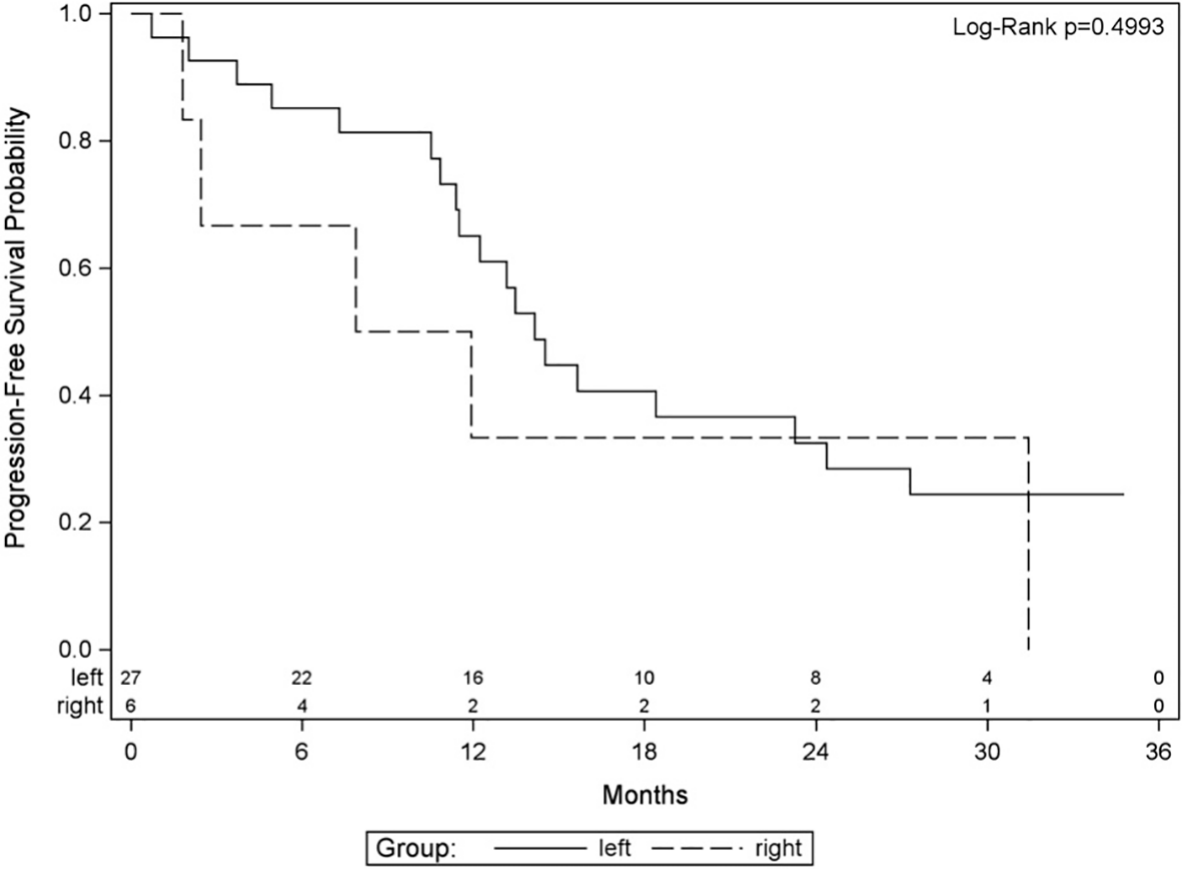
Kaplan–Meier plot for progression-free survival by tumor side.

**Figure 5:**
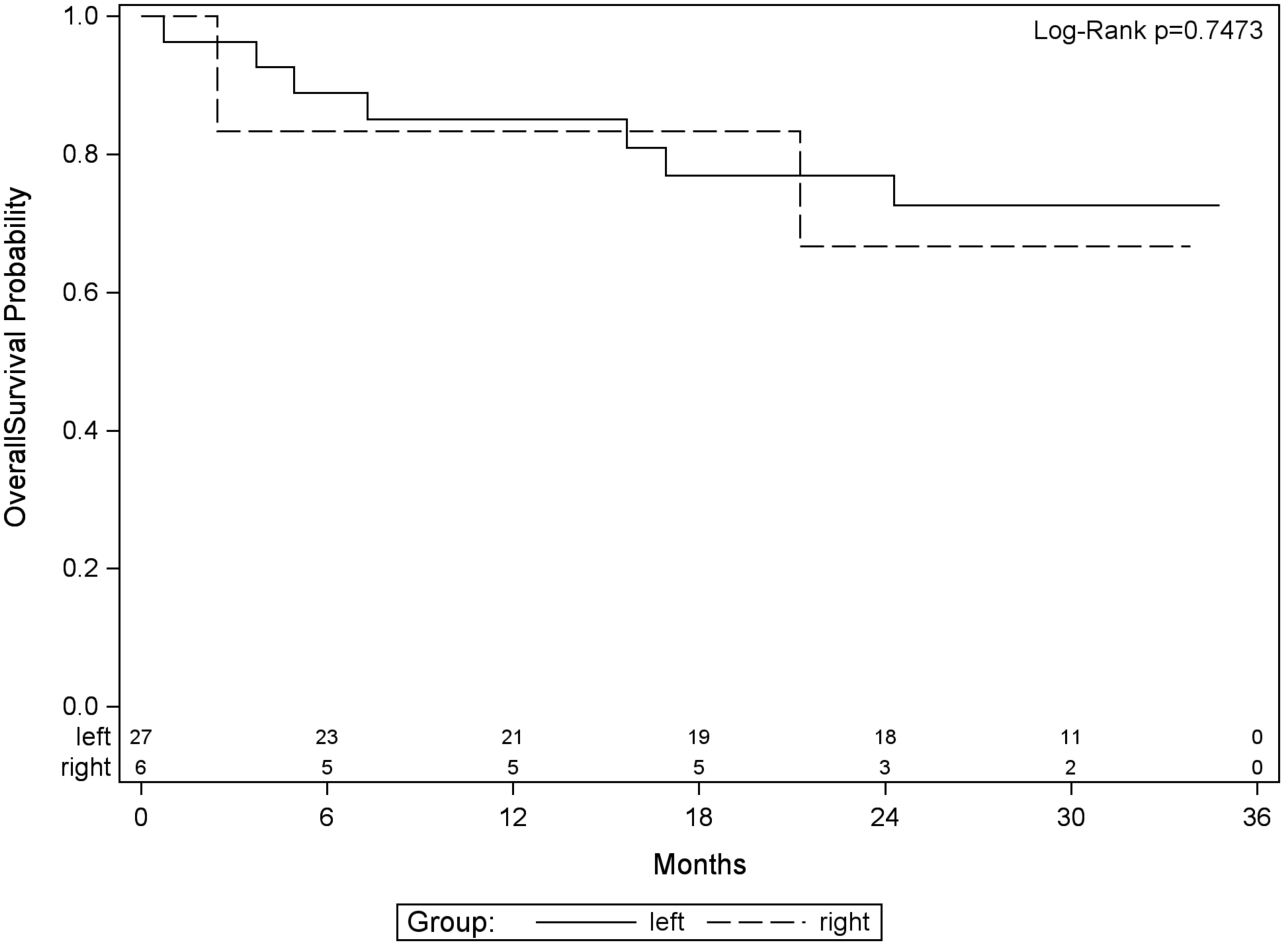
Kaplan–Meier plot for OS by tumor side.

Time to progression was defined as the time from registration date to the date of first observed progression including the detection of new lesions or progression of existing metastases. From 33 patients, 20 had progression and 13 had no documented progression until the end of the study. Median time without progression was 14.5 months. Twelve- and 24-month survival rates without progression were 62.4% and 34%, respectively. PFS was defined as the time from registration date to the date of first observed progression or death. From 35 patients, 26 patients had a PFS event, and nine patients had no event. First event was one secondary carcinoma, 20 patients had metastases progression as first event, and five patients died without previous progression. Median PFS was 13.2 months. Twelve- and 24-month PFS rates were 55.4% and 30.8%, respectively (**Figure 2**). OS was defined as the time from registration date to the date of death of any cause. Twelve- and 24- months OS rates were 85.6% and 73.3%, respectively (**Figure 3**). Kaplan–Meier analysis of PFS und OS by tumor side showed no statistical difference between left- and right-sided tumors (**Figures 4**, **5**) but showed a trend toward better outcome in left-sided tumors.

## Discussion

Long-term survival and cure are possible in patients with resectable CLM, leading to 5-year survival rates of 25%–40% if R0 resection is achieved (6–9). Upfront resection of resectable CLM is a therapeutic option for patients with limited CLM. In comparison to the beneficial effect of adjuvant chemotherapy in stage III CRC, the advantage of postoperative chemotherapy after curative resection of CLM is uncertain. However, only a few clinical trials were performed and the available data showed improvements in DFS but not in OS. Further studies investigated the use of perioperative chemotherapy in resectable CLM to enhance the outcome. This approach offers benefits, such as downsizing of liver metastases that enable less extensive surgery, elimination of potential micrometastases, the reduction of the risk of intrahepatic and extrahepatic recurrences, and the delineation of tumor biology. Perioperative chemotherapy with FOLFOX remains one of the standard treatments in patients with resectable CLM since presentation of the data from the EPOC study from the EORTC. In this trial, perioperative chemotherapy with FOLFOX and surgery was compared with surgery alone in CLM and demonstrated a significant better median PFS if perioperative chemotherapy was administered (11, 12). The OS showed a trend in favor of perioperative chemotherapy but was not statistically significant (median OS, 63.7 versus 55.0 months). Potential explanation was that the study was not designed or powered to detect differences in OS (17). In addition, more patients in the surgery alone group with disease progression received palliative chemotherapy as treatment when compared with patients in the perioperative chemotherapy group who progressed. This confounding variable could clearly affect OS but not PFS.

To improve this outcome, the addition of anti-EGFR antibodies in the perioperative setting is an interesting approach, as chemotherapy in combination with anti-EGFR antibodies improved DFS and OS in advanced disease and had the potential for curative resection in previously unresectable CLM (15, 20, 26–30). First-line FOLFIRI plus panitumumab was associated with favorable efficacy in patients with wild-type RAS. In a phase II study, the median OS was 26 months. Almost a quarter of patients with previously unresectable CLM became resectable after 8 weeks of therapy. The median OS in patients with and without metastasectomies was 40 and 22 months, respectively (31). In the adjuvant setting, the addition of anti-EGFR showed no benefit (32, 33). However, the probability of circulating tumor cells in metastatic disease is increased and justified the investigation of anti-EGFR in the perioperative setting in a prospective trial.

In this LM02 study, the preoperative administration of four cycles of panitumumab and FOLFIRI in patients with primary resectable RAS wild-type CLM resulted in a radiological ORR in two-thirds of patients with a manageable grade 3 diarrhea rate of 14.3% of patients. Surgery of liver metastases was done in 82.9% of patients. Two patients died after surgery because of hepatic failure. Both patients had a major liver surgery with hemihepatectomy right. Postoperative biliary complication was the reason for death in both patients. All eight cycles of postoperative therapy could be given to 60% of all patients who started postoperative treatment. Median PFS was 13.2 months, and the 24-month OS rate was 73.3%. In the New EPOC trial, a similar approach was investigated, but, as chemotherapy backbone, FOLFOX was mainly used. In the new EPOC randomized study, perioperative chemotherapy (FOLFOX, CAPOX, or FOLFIRI) with or without cetuximab was investigated in resectable CLM (34, 35). About two-thirds of patients received chemotherapy regimen one (FOLFOX), followed by regimen two (CAPOX) in a fifth and about 10% of patients received as chemotherapy FOLFIRI with or without cetuximab. About 73% and 76% of the patients in the chemotherapy alone group (CT group) and in the chemotherapy plus cetuximab group (CTX group) completed 12 weeks of preoperative therapy, respectively. CR or PR occurred in 61% of patients receiving chemotherapy alone and in 72% of patients receiving chemotherapy plus cetuximab. About 86% of patients were operated. Most patients had a R0 resection (82% in the CT group and 79% of the CTX group). In addition, 46% and 48% of patients in the CT group and in the CTX group completed 12 weeks of postoperative therapy, respectively. Unexpectedly, median PFS and median OS were better in the CT group compared with patients who received chemotherapy plus cetuximab (median PFS, 22.2 versus 15.5 months; median OS, 81.0 versus 55.5 months). Possible explanations included interactions between cetuximab and chemotherapy backbone (FOLFOX and CAPOX), further mutations in the EGFR pathway, and upregulation of alternative signaling pathways in combination with surgery. It has been proposed that the interaction between oxaliplatin and cetuximab is potentially negative because cetuximab may protect against free radical damage by platinum (36). Furthermore, patients with KRAS-mutations who were treated with an EGFR inhibitor had an inferior outcome in the oxaliplatin studies (15, 18) compared with patients in the irinotecan-based studies who had a similar outcome to chemotherapy-only patients (19). Subgroup analysis of the New EPOC trial showed that cetuximab in combination with the chemotherapy FOLFOX had a detrimental effect compared with patients receiving FOLFOX alone. However, patients receiving the chemotherapy FOLFIRI had a better PFS if cetuximab was added. However, this analysis should be interpreted cautiously due to the few patients receiving FOLFIRI as chemotherapy backbone in that study. Furthermore, in the updated analysis of the OS, the differences between the chemotherapy backbone were not confirmed.

To our knowledge, there has not been any other trial investigating preoperative panitumumab and FOLFIRI followed by liver resection and postoperative therapy with panitumumab and FOLFIRI in primary resectable CLM before. Unanswered questions in this patient population are the best chemotherapy combination with targeted agents to induce maximum response and the length of the initial treatment to verify response. The majority of available data are with oxaliplatin as chemotherapy. Treatment combination and number of preoperative cycles in the presenting LM02 trial are different from other trials that were done in the same indication. The EPOC trial administered six cycles of perioperative FOLFOX, and, in the New EPOC trial, patients received six cycles of FOLFOX/CAPOX/FOLFIRI with or without cetuximab compared with four cycles preoperative and eight cycles postoperative panitumumab and FOLFIRI in this LM02 trial. Despite the reduced numbers of preoperative therapies, our study achieved an ORR of 65.7% compared with 43% and 72% in the EPOC and New EPOC trial. Grade 3–4 preoperative toxicities were more common in the EPOC and New EPOC trial compared with our trial. The lower toxicity rate in our trial is contributed to the reduced number of preoperative cycles. However, diarrhea grade 3 was slightly higher in the LM02 trial with 11.4% compared with 9% in the New EPOC trial and 8% in the EPOC trial. The higher diarrhea rate is attributed to irinotecan as chemotherapy backbone and is a known side effect. One patient each died in the LM02 trial (sepsis) and in the New EPOC trial (cardiac arrest) during the preoperative phase, whereas, in the EPOC trial, no patient died. Postoperative two patients died in the LM02 trial compared with three deaths in the EPOC trial (two in the surgery-alone group and one in the chemotherapy group) and no deaths in the New EPOC trial. Median PFS was significant worse with the addition of cetuximab to chemotherapy in the New EPOC trial compared with chemotherapy alone (mPFS, 15.5 versus 22.2 months). In the LM02 trial, the mPFS was equally modest with 13.2 months.

Possible criticism of the LM02 trial is the small sample size of this phase II study. This combination therapy was not evaluated before as perioperative therapy in primary resectable CLMs. That is why our study group decided to plan a phase II study with a two-step design in a small sample size to evaluate efficacy and safety. At time of initiating of this study, there was only limited experience as conversion therapy of this combination in patients with primary unresectable CLMs. Furthermore, only 15 patients received this combination in the New EPOC trial. A further possible criticism is the continuation of the study despite the negative results of the New EPOC trial, which was extensively criticized for lack of adequate surgical quality control, imbalance in patients’ characteristics, variations in chemotherapy backbone, and increased rate of early death without clear attribution (37). First, the LM02 study was initiated, whereas the new EPOC study was still recruiting. Data from the New EPOC trial were published in April 2014 (34). At that time, the interim analysis of the LM02 trial was performed after 15 patients. The criteria for continuation of the LM02 study were met as predefined. We were encouraged by the high ORR in the interim analysis of 86.7% compared with the new EPOC study with 70% ORR in the cetuximab + chemotherapy arm. In addition, a subgroup analysis of the New EPOC trial favored the addition of cetuximab to the chemotherapy backbone FOLFIRI. The fact that we used FOLFIRI as chemotherapy backbone, the high ORR in the interim analysis, and the subgroup analysis of the New EPOC trial favoring cetuximab + FOLFIRI justified the continuation of the LM02 study. Nevertheless, the mPFS data in the LM02 study were equally modest as compared with that in the New EPOC trial.

## Conclusions

Panitumumab and FOLFIRI as perioperative therapy for resectable CLM result in a radiological ORR in 65.7% with a manageable grade 3 diarrhea rate. Median PFS was 13.2 months, and the 24-month OS rate was 73.3%. These data are insufficient to widen the indication of panitumumab from the unresectable setting to the setting of resectable CLM.

## Data availability statement

The original contributions presented in the study are included in the article/supplementary material. Further inquiries can be directed to the corresponding author.

## Ethics statement

The studies involving human participants were reviewed and approved by Ethik Komission Medizinische Universität Wien. The patients/participants provided their written informed consent to participate in this study.

## Author contributions

GP: Conceptualization, Investigation, writing – original draft, visualization, project administration; TG: Conceptualization, Investigation, project administration, Writing – Review and Editing; JT: Conceptualization, Review, project administration, Writing – Review and editing; IK: Investigation; KK: Investigation; FL: Investigation; AA: Investigation; WE: Investigation; RF: Investigation; JA: Investigation; AP: Investigation; JS: Investigation; LS: formal analysis; DÖ: Project administration, Investigation, Supervision, writing – Review and Editing. All authors contributed to the article and approved the submitted version.

## References

[1] Malvezzi M Carioli G Bertuccio P Rosso T Boffetta P Levi F. European cancer mortality predictions for the year 2016 with focus on leukaemias. Ann Oncol (2016) 27:725–31. doi: 10.1093/annonc/mdw022 26812903

[2] Siegel RL Miller KD Jemal A. Cancer statistics, 2017. CA: A Cancer J Clin (2017) 67:7–30. doi: 10.3322/caac.21387 28055103

[3] Midgley R Kerr D. Colorectal cancer. Lancet (1999) 353:391–9. doi: 10.1016/S0140-6736(98)07127-X 9950460

[4] Kindler HL Shulman KL. Metastatic colorectal cancer. Curr Treat Options Oncol (2001) 2:459–71. doi: 10.1007/s11864-001-0068-7 12057092

[5] Chong G Cunningham D. Improving long-term outcomes for patients with liver metastases from colorectal cancer. J Clin Oncol (2005) 23:9063–6. doi: 10.1200/JCO.2005.04.4669 16301589

[6] Fong Y Fortner J Sun RL Brennan MF Blumgart LH. Clinical score for predicting recurrence after hepatic resection for metastatic colorectal cancer: analysis of 1001 consecutive cases. Ann Surg (1999) 230:309–21. doi: 10.1097/00000658-199909000-00004 PMC1420876 10493478

[7] Choti MA Sitzmann JV Tiburi MF Sumetchotimetha W Rangsin R Schulick RD. Trends in long-term survival following liver resection for hepatic colorectal metastases. Ann Surg (2002) 235:759–66. doi: 10.1097/00000658-200206000-00002 PMC1422504 12035031

[8] Fong Y Cohen AM Fortner JG Enker WE Turnbull AD Coit DG. Liver resection for colorectal metastases. J Clin Oncol (1997) 15:938–46. doi: 10.1200/JCO.1997.15.3.938 9060531

[9] Kanas GP Taylor A Primrose JN Langeberg WJ Kelsh MA Mowat FS. Survival after liver resection in metatatic colorectal cancer: review and meta-analysis of prognostic factors. Clin Epidemiol (2021) 4:283–301. doi: 10.2147/CLEP.S34285 PMC3496330 23152705

[10] Zakaria S Donohue JH Que FG Farnell MB Schleck CD Ilstrup DM. Hepatic resection of colorectal metastases; value for risk scoring systems? Ann Surg (2007) 246:183–91. doi: 10.1097/SLA.0b013e3180603039 PMC1933577 17667495

[11] Nordlinger B Sorbye H Glimelius B Poston GJ Schlag PM Rougier P. Perioperative chemotherapy with FOLFOX4 and surgery versus surgery alone for resectable liver metastases from colorectal cancer (EORTC Intergroup trial 40983): a randomised controlled trial. Lancet (2008) 371:1007–16. doi: 10.1016/S0140-6736(08)60455-9 PMC2277487 18358928

[12] Nordlinger B Sorbye H Glimelius B Poston GJ Schlag PM Rougier P. Perioperative FOLFOX4 chemotherapy and surgery versus surgery alone for resectable liver metastases from colorectal cancer (EORTC 40983): long term results of a randomised, controlled, phase 3 trial. Lancet Oncol (2013) 14:1208–15. doi: 10.1016/S1470-2045(13)70447-9 24120480

[13] Wang ZM Chen YY Chen FF Wang SY Xiong B. Peri-operative chemotherapy for patients with resectable colorectal hepatic metastasis: a meta-analysis. Eur J Surg (2015) 41:1197–203. doi: 10.1016/j.ejso.2015.05.020 26094113

[14] Peeters M Price TJ Cervantes A Sobrero AF Ducreux M Hotko Y. Randomized phase III study of panitumumab with FOLFIRI compared with FOLFIRI alone as second line treatment in patients with metastatic colorectal cancer. J Clin Oncol (2010) 28:4706–13. doi: 10.1200/JCO.2009.27.6055 20921462

[15] Douillard JY Oliner KS Siena S Tabernero J Burkes R Barugel M. Panitumumab-FOLFOX4 treatment and RAS mutations in colorectal cancer. N Engl J Med (2013) 369:1023–34. doi: 10.1056/NEJMoa1305275 24024839

[16] Peeters M Oliner KS Price TJ Cervantes A Sobrero AF Ducreux M. Analysis of KRAS/NRAS mutations in a phase III study of panitumumab with FOLFIRI compared with FOLFIRI alone as second-line treatment for metastastic colorectal cancer. Clin Cancer Res (2015) 21:5469–79. doi: 10.1158/1078-0432.CCR-15-0526 26341920

[17] Padmanabhan C Parikh A. Perioperative chemotherapy for resectable colorectal hepatic metastases – What does the EORTC 40983 trial update mean? Hepatobiliary Surg Nutr (2015) 4:80–3. doi: 10.3978/j.issn.2304-3881.2014.08.05 PMC4318966 25713808

[18] Bokemeyer C Bondarenko I Hartmann JT de Braud F Schuck G Zubel A. Efficacy according to biomarker status of cetuximab plus FOLFOX-4 as first-line treatment for metastatic colorectal cancer: the OPUS study. Ann Oncol (2011) 22:1535–46. doi: 10.1093/annonc/mdq632 21228335

[19] Van Cutsem E Köhne CH Lang I Folprecht G Nowacki MP Cascinu S. Cetuximab plus irinotecan, fluorouracil, and leucovorin as first-line treatment for metastatic colorectal cancer: updated analysis of overall survival according to tumor KRAS and BRAF mutation status. J Clin Oncol (2011) 29:2011–9. doi: 10.1200/JCO.2010.33.5091 21502544

[20] Douillard JY Siena S Cassidy J Tabernero J Burkes R Barugel M. Final results from PRIME: randomized phase III study of panitumumab with FOLFOX4 for first-line treatment of metastatic colorectal cancer. Ann Oncol (2014) 25:1346–55. doi: 10.1093/annonc/mdu141 24718886

[21] Schwartzberg L Rivera F Karthaus M Gianpiero F Canon JL Hecht JR. Analysis of KRAS/NRAS mutations in PEAK: a randomized phase 2 study of FOLFOX6 + panitumumab or bevacizumab as 1st line treatment for wild type KRAS (exon 2) metastatic colorectal cancer (2013). ASCO, Chicago, IL (Accessed May 31 – June 4, 2013).

[22] Patterson SD Peeters M Siena S Van Cutsem E Humblet Y Van Laethem JL. Comprehensive Analysis of KRAS and NRAS mutations as predicitive biomarkers for sinlge agent panitumumab response in a randomized, phase 3 metatatic colorectal cancer study (20020408) (2013). ASCO Chicago, IL (Accessed May 31 – June 4, 2013).

[23] Dindo D Demartines N Clavien PA. Classification of surgical complications. Ann Surg (2004) 240:205–13. doi: 10.1097/01.sla.0000133083.54934.ae PMC1360123 15273542

[24] Rubbia-Brandt L Giostra E Brezault C Roth AD Andres A Audard V. Importance of histological tumor response assessment in predicting the outcome in patients with colorectal liver metastases treated with neoadjuvant chemotherapy followed by liver surgery. Ann Oncol (2007) 18:299–304. doi: 10.1093/annonc/mdl386 17060484

[25] National Cancer Institute: Common Terminology Criteria for Adverse Events (CTCAE) version 4.0 (2009) (Accessed May 28, 2009).

[26] Van Cutsem E Köhne C-H Hitre E Zaluski J Chien CC Makhson A. Cetuximab and chemotherapy as initial treatment for metastatic colorectal cancer. N Engl J Med (2009) 360:1408–17. doi: 10.1056/NEJMoa0805019 19339720

[27] Bokemeyer C Bondarenko I Makhson A Hartmann JT Aparicio J de Braud F. Fluorouracil, leucovorin, and oxaliplatin with and without cetuximab in the first-line treatment of metastatic colorectal cancer. J Clin Oncol (2009) 27:663–71. doi: 10.1200/JCO.2008.20.8397 19114683

[28] Folprecht G Gruenberger T Bechstein WO Raab HR Lordick F Hartmann JT. Tumour response and secondary resectability of colorectal liver metastases following neoadjuvant chemotherapy with cetuximab: the CELIM randomised phase 2 trial. Lancet Oncol (2010) 11:38–47. doi: 10.1016/S1470-2045(09)70330-4 19942479

[29] Maughan TS Adams RA Smith CG Meade AM Seymour MT Wilson RH. Addition of cetuximab to oxaliplatin-based first-line combination chemotherapy for treatment of advanced colorectal cancer: results of the randomised phase 3 MRC COIN trial. Lancet (2011) 377:2103–14. doi: 10.1016/S0140-6736(11)60613-2 PMC3159415 21641636

[30] Peeters M Price TJ Cervantes A Sobrero AF Ducreux M Hotko Y. Final results from a randomized phase 3 study of FOLFIRI ± panitumumab for second-line treatment of metastatic colorectal cancer. Ann Oncol (2014) 25:107–16. doi: 10.1093/annonc/mdt523 24356622

[31] Geredeli C Yasar N. FOLFIRI plus panitumumab in the treatment of wild-type KRAS and wild-type NRAS metastatic colorectal cancer. World J Surg Oncol (2018) 16:67. doi: 10.1186/s12957-018-1359-9 29587749 PMC5870197

[32] Alberts SR Sargent DJ Nair S Mahoney MR Mooney M Thibodeau SN. Effect of oxaliplatin, fluorouracil, and leucovorin with or without cetuximab on survival among patients with resected stage III colon cancer: a randomized trial. JAMA (2012) 307:1383–93. doi: 10.1001/jama.2012.385 PMC3442260 22474202

[33] Taieb J Tabernero J Min E Subtil F Folprecht G Van Laethem JL. Oxaliplatin, fluorouracil, and leucovorin with or without cetuximab in patients with resected stage III colon cancer (PETACC-8): an open-label, randomised phase 3 trial. Lancet Oncol (2014) 15:862–73. doi: 10.1016/S1470-2045(14)70227-X 24928083

[34] Primose J Falk S Finch-Jones M Valle J O’Reilly D Siriwardena A. Systemic chemotherapy with or without cetuximab in patients with resectable colorectal liver metastasis: the New EPOC randomised controlled trial. Lancet Oncol (2014) 15:601–11. doi: 10.1016/S1470-2045(14)70105-6 24717919

[35] Bridgewater JA Pugh SA Maishman T Eminton Z Mellor J Whitehead A. Systemic chemotherapy with or without cetuximab in patients with resectable colorectal liver metastasis (New EPOC): long-term results of a multicentre, randomised, controlled, phase 3 trial. Lancet Oncol (2020) 21:398–411. doi: 10.1016/S1470-2045(19)30798-3 32014119 PMC7052737

[36] Dahan L Sadok A Formento JL Seitz JF Kovacic H. Modulation of cellular redox state underlies antagonism between oxaliplatin and cetuximab in human colorectal cancer cell lines. Br J Pharmacol (2009) 158:610–20. doi: 10.1111/j.1476-5381.2009.00341.x PMC2757701 19732064

[37] Nordlinger B Poston GJ Goldberg RM. Should the results of the new EPOC trial change practice in the management of patients with resectable metastastic colorectal cancer confined to the liver? JCO (2015) 33(3):241–3. doi: 10.1200/JCO.2014.58.3989 25403221

